# Smokeless Tobacco Point of Sale Advertising, Placement and Promotion: Associations With Store and Neighborhood Characteristics

**DOI:** 10.3389/fpubh.2021.668642

**Published:** 2021-05-13

**Authors:** Shirley A. James, John G. Heller, Chantel J. Hartman, Andrew C. Schaff, Nasir Mushtaq, Laura A. Beebe

**Affiliations:** ^1^Department of Biostatistics and Epidemiology, Hudson College of Public Health, University of Oklahoma Health Sciences Center, Oklahoma City, OK, United States; ^2^Center for Chronic Disease Prevention and Health Promotion, Oklahoma State Department of Health, Oklahoma City, OK, United States

**Keywords:** tobacco retail, smokeless tobacco, smokeless tobacco advertising, point of sale study, tobacco products

## Abstract

**Introduction:** Objectives of this study were to determine retail and neighborhood characteristics associated with smokeless tobacco (ST) product promotion, price promotion, and storefront advertising among retailers in Oklahoma.

**Methods:** In this statewide point-of-sale study, we collected data from 1,354 ST retailers. Using store characteristics and census tract information, we estimated summary statistics and adjusted prevalence ratios during 2019–2020.

**Results:** Of ST retailers audited, 11.0% demonstrated ST youth promotion, 43.0% ST price promotions, and 19.6% ST storefront advertising. The adjusted prevalence ratio (aPR) for convenience stores was higher for all three ST strategies: youth promotion (aPR = 3.4, 95% CI 1.9, 6.2), price promotion (aPR = 3.8, 95% CI 2.9, 5.0), and storefront advertising (aPR=16.4, 95% CI 6.7, 40.3) compared to other store types. Metropolitan tobacco retailers had higher aPRs for youth promotion (aPR = 1.7, 95% CI 1.12 2.6) and storefront advertising (aPR = 1.5, 95% CI 1.2, 1.9).

**Conclusions:** Findings of this study suggest there are currently ample opportunities for youth and adults at risk for tobacco initiation to be exposed to ST products in the retail environment. Convenience stores, more likely to be found and utilized in rural areas compared to metropolitan areas, are disproportionately more likely to engage in marketing strategies that could lure youth into trying smokeless tobacco.

## Background and Purpose

Smokeless tobacco (ST) use can lead to nicotine addiction, is associated with cancers of the oral cavity, esophagus, and pancreas, and is associated with higher levels of mortality from both heart disease and stroke (1–8). ST products are growing in popularity, partially because they can be used indoors in places where smoking is not allowed (9, 10). The Youth Risk Behavior Surveillance System data (2018) report that 4.2% of youth in the US, and more specifically, 6.8% of Oklahoma youth use ST, meaning they used chewing tobacco, snuff, dip, snus, or dissolvable tobacco on one or more of the 30 days before participation in a survey about this topic (11, 12). Worldwide, at least 303 million people (5.6% of the population) 15 years and older are current ST users (13). This high level of ST use is a disturbing trend because not only has ST use proven harmful (1–8), it can serve as a tobacco initiation product (14).

The tobacco retail environment been linked to growth in tobacco use, but tobacco product advertising further normalizes its use in the community (15–18). Tobacco product advertising, promotion, and placement can all influence youth to start using tobacco at younger ages, and at increased rates (16, 18, 19). The tobacco industry continues to advertise heavily at retail outlets near schools and playgrounds, using storefront tobacco ads in locations where youth readily notice them (16–20). Total combined tobacco expenditures for outdoor tobacco advertising grew from 1.7 million dollars in 2016 to 1.8 million dollars in 2017 (18). One study reported 5.2% of retail store advertising involved ST products (18). The number and location of tobacco retail outlets in a community, specifically in proximity to schools, also increases the availability of tobacco products, the likelihood of tobacco experimentation among minors (19), and the sale of tobacco to minors (21). Studies have demonstrated a higher density of tobacco retailers in communities at greatest risk; those with low income, and with higher proportions of non-whites (18, 19, 22–25).

Price promotions are an effective vehicle for youth initiation. RJ Reynolds and Phillip Morris tobacco companies first recognized them as such in the 1980s (26). Stores entice both adults and youth to try ST with price promotion and with cross product price promotion with products including cigarettes (18). We defined cross product promotions in this study as situations in which retailers either offered a discount on cigarettes with the purchase of ST products, or when retailers offered a discount on ST products with the purchase of cigarettes. Additionally, retailers place tobacco products and advertising lower to the floor and within reach of products that appeal to youth (20), including soda and candy products, ICEE^®^ drinks, jewelry, and electronic merchandise (20, 24). Point-of-sale (POS) advertisements, which are advertisements displayed or distributed at retail locations, can introduce youth to tobacco products (18, 22). Assessments of the tobacco retail environment are an essential component in building awareness and documenting tobacco industry activity in communities. Previous POS studies have provided relevant information about both retail environments and attempts by tobacco companies to influence youth (18–20, 23–25).

In the rapidly evolving retail environment, information about ST product placement and advertising is informative but scarce, with the exception of the study by Widome et al. in 2012 (25). Our study contributes a unique perspective on the retail landscape for ST products by including a large statewide sample of retailers within a state with high tobacco prevalence including ST use. The aims of our study are to identify the prevalence of the following retailer characteristics, as well as their association with neighborhood census tract characteristics: retailer type, storefront ST product advertisements, ST price promotions or cross-product promotions with cigarettes, and promotion of ST products to youth.

## Methods

This study was completed in a partnership between the University of Oklahoma Health Sciences Center's Hudson College of Public Health, and the Oklahoma State Department of Health's (OSDH) Center for Chronic Disease Prevention and Health Promotion. The program is entitled “Project CHAT: Combatting Heavy Advertisement of Tobacco initiative.”

### Training and Sample

Project staff and OSDH partners attended a 2-day Standardized Tobacco Assessment for Retail Settings (STARS) training provided by Counter Tools. The STARS surveillance tool was designed for practitioners to inform tobacco control policies for the point of sale, and was developed from collaboration by state and community tobacco control researchers from five state health departments, the CDC, and the Public Health Law Center (formerly the Tobacco Control Legal Consortium). Training for this study included a field exercise to practice store assessments and data collection with the STARS tool, which can be found here https://countertobacco.org/resources-tools/store-assessment-tools/stars/. The Oklahoma Department of Mental Health and Substance Abuse Services provided a list of licensed tobacco retailers used during the Synar Purchase Survey. Eligible tobacco and cigarette licensed retailers for this study included convenience stores, drug stores, pharmacies, grocery stores, and mass merchandisers. Retailers with less potential for exposure to youth were excluded. These included liquor stores, bars, tobacco shops, tribal-owned smoke shops, hookah lounges and vape shops, where those under 18 years of age are not legally allowed to enter. Free-standing vape shops are not licensed as tobacco retailers in Oklahoma; thus, they were not included in the sampling frame. From the list of 3,650 eligible licensed tobacco retailers, a random sample to be audited (*n* = 1,713) was selected proportional to the number of stores in each county. The number of stores selected per county ranged from 5 to 90.

### Store Audits

Project staff completed 1,560 tobacco retail audits between March and December of 2017 using the STARS form and protocol. Four trained project staff collected retailer data via the Store Audit Center, the online platform created by Counter Tools. The 16-item STARS tool included questions about store type, exterior and interior advertising, type of tobacco products sold, and price, placement, and promotion of tobacco products (22).

Whether or not the store accepted Special Supplemental Nutrition Program for Women, Infants, and Children (WIC) benefits was included as a covariate. Store audit data were also used to define the outcomes for this study. These included presence of storefront ST advertisements, presence of ST price promotions, or cross promotions with cigarettes. Cross promotions occurred when retailers offered reduced prices on cigarettes with the purchase of ST. ST youth promotion occurred when ST products or advertisements were placed within three feet of the floor or 12 inches from products appealing to children.

### Neighborhood Characteristics

Tobacco retailer addresses were geocoded using ArcGIS (20) software. The 2010 US census tract geoids were used to characterize the neighborhood demographic variables for each retail location. These included the percent African American race, percent Hispanic ethnicity, percent Native American race, percent living at or below poverty level, percent whose highest education was high school or GED or less, and percent under 18 years of age. Using data for the entire State of Oklahoma, we established quartiles for these census tract variables to allow us to compare retailers in the highest quartile to retailers in the lower three quartiles for each of these variables. The top quartile cut-points included: HS education/GED or less = 58.8%; at or below poverty level = 24.7%; <18 years of age = 39.1%; African-American race = 8.6%; American Indian race= 10.1%; Hispanic ethnicity = 11.5% (Tables 1, 2). Rural-Urban Commuting area codes (RUCA) were used to identify retailer location as urban or rural. RUCA codes zero through six represented urban areas, and RUCA codes seven and up represented smaller towns and rural areas. ArcGIS (20) was used to determine whether or not the tobacco retailer was within one mile of a high school or a middle school.

**Table 1:** Characteristics of audited tobacco retailers selling smokeless tobacco (ST) products (*n* = 1,354).

	***N***	**%**
**Store characteristics**		
Store type		
Convenience store	1,031	76.1
• Drug store, pharmacy, grocery, or mass merchandiser	323	23.9
Accepts the Special Supplemental Nutrition Program for Women, Infants, and Children (WIC)	448	33.2
**Neighborhood characteristics**		
Metropolitan (urban) community	805	59.5
High school or middle school within one mile (5,280 feet)	763	56.4
% population with high school education or less		
Q4: >58.8%	463	34.2
Q1–3: 0–58.8%	891	65.8
% population at or below poverty level		
Q4: >24.7%	909	67.1
Q1–3: 0–24.7%	445	32.9
% children/youth		
Q4: >39.1%	311	23.0
Q1–3: 0–39.1%	1,043	77.0
% Black, non-Hispanic		
Q4: >8.6%	199	14.7
Q1–3: 0–8.6%	1,155	85.3
% American Indian, non-Hispanic		
Q4: >10.1%	467	34.5
Q1–3: 0–10.1%	887	65.5
% Hispanic or Latino		
Q4: >11.5%	299	22.1
Q1–3: 0–11.5%	1055	77.9
**Smokeless tobacco (ST) product placement & promotion**		
Exterior ST advertising	265	19.6
Youth Promotion (any)	149	11.0
ST products placed within 12 inches of youth-related items	66	4.9
ST advertisements within 3 feet of floor	100	7.4
Price promotion (any)	582	43.0
ST price promotions	580	42.9
ST cross-product promotions with cigarettes	4	0.3

**Table 2:** Prevalence of study outcomes and adjusted prevalence ratios (95% CI) by retailer and neighborhood characteristic (*n* = 1,354).

	**Youth promotion**		**Price promotion**		**Exterior ST Advertising**	
	**Percent**	**Adjusted prevalence ratio (95% CI)**	**Percent**	**Adjusted prevalence ratio (95% CI)**	**Percent**	**Adjusted prevalence ratio (95% CI)**
**Store characteristics**						
Store type						
Convenience store	12.8	**3.41 (1.86, 6.23)**	51.7	**3.80 (2.87, 5.03)**	25.3	**16.38 (6.66, 40.28)**
Drug store, pharmacy, grocery, or mass	5.3	Ref	15.2	Ref	1.6	Ref
Merchandiser						
Accepts WIC						
Yes	9.4	1.28 (0.82, 2.01)	32.6	1.11 (0.97, 1.28)	10.8	1.00 (0.76, 1.33)
No	11.9	Ref	48.3	Ref	24.1	Ref
**Neighborhood characteristics**						
Population density						
Metropolitan	13.3	**1.74 (1.19, 2.55)**	40.1	0.87 (0.77, 0.99)	22.9	**1.54 (1.22, 1.95)**
Small town/rural	7.7	Ref	47.2	Ref	14.8	Ref
High school or middle school within one mile (5,280 feet)						
Yes	11.9	1.31 (0.91, 1.89)	43.5	1.06 (0.94, 1.20)	18.9	0.98 (0.80, 1.21)
No	9.8	Ref	42.3	Ref	20.6	Ref
% population with high school education or less						
Q4: >58.8%	10.6	0.97 (0.64, 1.46)	46.9	1.02 (0.89, 1.17)	21.4	1.12 (0.89, 1.41)
Q1–3: 0–58.8%	11.2	Ref	41	Ref	18.7	Ref
% population at or below poverty level						
Q4: >24.7%	10.3	0.83 (0.55, 1.26)	45.3	**1.18 (1.02, 1.38)**	20.2	1.13 (0.89, 1.44)
Q1–3: 0–24.7%	12.4	Ref	38.2	**Ref**	18.5	Ref
% children/youth						
Q4: >39.1%	14.5	1.46 (0.98, 2.19)	43.4	1.08 (0.93, 1.26)	20.9	1.07 (0.84, 1.36)
Q1–3: 0–39.1%	10	Ref	42.9	Ref	19.2	Ref
% Black, non-Hispanic						
Q4: >8.6%	16.6	1.51 (0.96, 2.37)	44.7	1.00 (0.84, 1.18)	29.8	**1.40 (1.09, 1.80)**
Q1–3: 0–8.6%	10	Ref	42.7	Ref	17.9	Ref
% American Indian, non-Hispanic						
Q4: >10.1%	8.8	0.84 (0.56, 1.26)	17.3	0.98 (0.86, 1.13)	16.3	0.80 (0.63, 1.02)
Q1–3: 0–10.1%	12.2	Ref	30	Ref	21.4	Ref
% Hispanic or Latino						
Q4: >11.5%	14.4	1.27 (0.81, 2.01)	46.5	1.04 (0.89, 1.21)	22.1	0.96 (0.74, 1.23)
Q1–3: 0–11.5%	10	Ref	42	Ref	18.9	Ref

*Bolded values indicate significant at or below the alpha = 0.05 level*.

### Statistical Analysis

We downloaded our tobacco retailer data from our Store Audit Center, cleaned it, and checked it for consistency. We calculated descriptive statistics for all dependent and independent variables associated with our sample of retailers. Using percentages, we then reported the prevalence of each outcome variable within each level of independent variable. Finally, we examined relationships between each of the three outcome variables and the covariates described above using adjusted prevalence ratios. To account for clusters of stores within each census tract, which ranged from one to seven, we used generalized estimating equation (GEE) analysis with PROC GENMOD, adding the census tract variable to the repeated statement. PROC GENMOD offers logistic Poisson regression capabilities with robust error variances (21) and produces prevalence proportion ratios (PPRs) and 95% CIs to describe associations. SAS version 9.4 was used to analyze data during 2019–2020. The University of Oklahoma Institutional Review Board determined this study was not human subject research, thus needed no approval (OUHSC IRB reference #7679).

## Results

Statewide, 91% of our random sample of tobacco retailers were successfully audited, resulting in 1,560 completed audits. A majority of the retailers that were not audited did not exist or could not be found (64%, *n* = 89), largely due to address errors within the list. An additional 14% (*n* = 20) had permanently closed since the list was created. Of the retailers audited, the 1,354 that sold ST products were included in this study (87%).

Three-quarters (76%) of retailers selling ST products were convenience stores, and one-third (33%) accepted Supplemental Nutrition Program for Women, Infants, and Children (WIC) benefits. Almost two-thirds (60%) were located in metropolitan communities, and more than half (56%) were located within one mile of a high school or middle school (Table 1).

Of retailers included in this analysis, one-fifth (20%) had ST storefront advertisements, and less than one tenth had ST products placed within one foot of youth related items (5%), or ST advertisements or products within three feet of the floor (7%). While almost half (43%) had ST price promotions, <1% (0.3%) had ST cross product promotions with cigarettes (Table 1).

Approximately one third of retailers (34%) were located in census tracts in which the population was in the top quartile for having a high school education or less, and more than one fifth (23%) were located in the top quartile for having children under the age of 18 years. Additionally, two thirds of retailers (67%) were located in census tracts in the highest quartile for living in poverty as defined by the US Census Bureau (22). More than one tenth were located in census tracts in the highest quartile for percentage of Black race (15%), more than one third were in census tracts in the highest quartile for the percentage of American Indian race (35%), and one fifth (22%) were in census tracts with the highest percentage of Hispanic or Latino ethnicity (Table 1).

### Factors Associated With Youth Promotion

In this study, we defined youth promotion of ST products as placement of ST products within 12 inches of items that appeal to children or youth, or ST advertisements within three feet of the floor. After adjusting for other variables in the model, the prevalence of ST youth promotion was almost three and one half times higher (aPR = 3.4 with 95% CI = 1.9, 6.2) for convenience stores as compared to drug stores, pharmacies, grocery stores, or mass merchandisers (13 vs. 5% of stores). The aPR was 1.7 times higher for metropolitan areas (95% CI = 1.2, 2.6) compared to rural areas (13 vs. 8% of stores) (Table 2).

### Factors Associated With ST Price Promotion or Cross Promotion With Cigarettes

After adjusting for other variables in the model, the prevalence of ST price promotions or cross promotions with cigarettes was almost four times higher in convenience stores compared to others (aPR = 3.8 with 95% CI = 2.9, 5.0). The adjusted prevalence ratio for price promotions or cross promotions with cigarettes was slightly higher when retailers were located in census tracts in which the percentage of the population at or below poverty level was in the top quartile (aPR = 1.2 with 95% CI = 1.02, 1.4) compared to census tracts in which the percentage was in the lower three quartiles (Table 2).

### Factors Associated With Outside ST Advertising

After adjusting for other variables in the model, the prevalence ratio for ST outside advertising was more than 16 times higher (aPR = 16.4 with 95% CI = 6.7, 40.3) for convenience stores as compared to drug stores, pharmacies, grocery stores, or mass merchandisers (25 vs. 2% of stores). Retailers in metropolitan areas also had a statistically higher prevalence of exterior ST advertising when compared to those in rural areas (aPR = 1.5 with 95% CI = 1.2, 2.0). Additionally, the prevalence ratio for outside ST advertising was almost one and a half (aPR = 1.4 with 95% CI = 1.1, 1.8) times higher for retailers in census tracts in which the percentage of the population reporting African American race was in the top 25th percentile for the state of Oklahoma compared to retailers in the lower percentiles (30 vs. 18% of stores) (Table 2).

## Discussion

Although cigarette and vapor product use among youth dominate discussions around the influence of POS strategies, in this study we found ST advertising and product placement also likely target youth. One-fifth of retailers involved in this study displayed storefront ST advertisements. ST price promotions were common among retailers, with almost half enticing consumers to purchase these products by offering them at a reduced rate. Another factor making the use of ST products in Oklahoma appealing is their exclusion from the most recent Oklahoma tobacco tax increase, which was $1.00 on other tobacco products. There is an upsurge in new ST products, which make them popular, as well as financially accessible, among youth. Studies involving the marketing of these products are vital to a complete understanding of ST product and advertising landscapes, especially given their attraction to young people (15–18, 20, 27–31).

The first aim of this study was to examine the association between retail and neighborhood characteristics associated with efforts to promote ST products to youth. While this placement was not common, in 66 stores (5%), ST products were placed within 12 inches of products that appeal to youth; in 100 stores (7%), ST products or promotions were placed within three feet of the floor, easily accessible to children and youth. This compares similarly to Widome and associates (25) who found 3% of stores had ST advertising less than three feet from the ground and 12% of stores had ads <12 inches from candy and snacks. Youth targeting was noted in a New York City study by Giovenco and associates, who found half of cigar and ENDS ads, and one third of cigarette and ST ads were placed on the main entry door. Over one third of all tobacco ads were placed at a height lower than three feet, and one quarter of cigar and cigarette ads were adjacent to sugary drink ads (32). This type of ST youth promotion is in violation of the intent of the Federal Cigarette Labeling and Advertising Act, applicable to any tobacco product (32).

Strongly associated with youth promotion was store type and location. As with other studies (32), convenience stores were more than three times more likely to demonstrate ST youth promotion than grocery and drug stores. Interestingly, in our study metropolitan areas were one and a half times more likely to demonstrate ST youth promotion than rural areas, a finding not replicated in other studies (16).

The second aim of this study was to examine the association between retail and neighborhood characteristics associated with ST price promotions, or cross promotions with cigarettes. First, our study conclusively demonstrated these promotions exist, with almost half of stores (43%, *n* = 580) having price promotions for ST products. Only four stores had cross promotions with cigarettes (0.3%). Secondly, there was an association with store type, with convenience stores almost four times more likely to have ST price promotions. In this study, ST price promotions specifically targeted those living in census tracts with a higher concentration of individuals living at or below the poverty level. As demonstrated in the literature, this is a dangerous trend, as price promotions have consistently targeted this group, raising the frequency of unplanned tobacco product purchases. Additionally, many price promotions effectively enhance youth initiation among children and teen shoppers (15, 28–30, 33, 34).

The third aim of this study was to examine the association between retail and neighborhood characteristics associated with storefront ST product advertisements (32). Our study found storefront ST advertising common, with one fifth of stores (*n* = 265) displaying ST advertising in places where any member of the public, but especially children and young people, can freely pass. More than half (56%, *n* = 763) of the stores in this study had a high school or a middle school within one mile, making exposure and access likely based on geographic proximity.

Store type was highly associated with storefront ST advertising, similarly to the study by Widome and associates (25), with convenience stores being more than 16 times more likely to have exterior ST advertisements than mass merchandisers, drug, or grocery stores. Similarly to Roberts and associates' findings (24), our study found a small but significant association between ST advertising in urban or metropolitan vs. more rural areas, and reported evidence of differential tobacco marketing at the point-of-sale, which disproportionately targeted urban and African American communities (22). This is especially troublesome, given the large percentage of convenience stores available to youth in metropolitan areas. Giovenco and associates' compared the percentage of retailers displaying storefront ST ads to those of other tobacco products in New York City; cigarette advertising was present in 40%, cigar in 27%, ENDS in 28%, and ST in 5% of retailers audited (32). Interestingly, exterior ST advertising was the only retailer characteristic significantly associated with census tracts in the highest quartile for percent African American, a finding not replicated in other studies.

Our study provides evidence for limiting outside and POS strategies, as they play an important role in youth ST exposure, access and initiation. Historically, banning outside advertising and POS displays has had a positive impact on tobacco use. In one study, having a POS display ban reduced overall adult daily smoking, male smoking and female smoking 7, 6, and 9%, respectively (26). Convenience stores, more likely to be found and utilized in rural areas compared to metropolitan areas, are disproportionately more likely to engage in marketing strategies that could lure youth into trying smokeless tobacco.

## Limitations

While this study was particularly robust given its representation of the entire state of Oklahoma, it has several limitations. First, as a cross-sectional study, we are capturing a snapshot of the ST retail landscape, and cannot make any causal inferences. Second, we are only reporting ST promotion in this study, and cigarette and particularly electronic nicotine delivery device results may be quite different. Analysis of some of our subgroups are limited by sample size. This is especially true with our storefront exterior advertising by store type (Table 2), and has resulted in a wide CI for this category. Finally, as with any POS study, the atmosphere involved in capturing this information is at times challenging, resulting in some incomplete data, as well as potential misclassification from auditor error. Most relevant to ST use, this study did not differentiate ST products from “General Snus,” as data collection occurred before the FDA regulation change.

## Data Availability Statement

The raw data supporting the conclusions of this article will be made available by the authors, without undue reservation.

## Author Contributions

SJ analyzed the data and wrote the paper. JH and AS collected data, assisted with data analysis, and did manuscript revisions. CH initiated the project and assisted with all portions of the project. NM assisted with data analysis, and did manuscript revisions. LB was in charge of this project from initiation to completion. Tasks included overseeing the project, supervising staff, analyzing data, and supervising manuscript preparation. All authors contributed to the article and approved the submitted version.

## Conflict of Interest

The authors declare that the research was conducted in the absence of any commercial or financial relationships that could be construed as a potential conflict of interest.
